# Bevacizumab as an Adjunct to Trabeculectomy in Primary Open-Angle Glaucoma: A Randomized Trial

**DOI:** 10.1155/2020/8359398

**Published:** 2020-01-31

**Authors:** Alper Bilgic, Aditya Sudhalkar, Anand Sudhalkar, Megha Trivedi, Viraj Vasavada, Shail Vasavada, Vaishali Vasavada, Samaresh Srivastava

**Affiliations:** ^1^Raghudeep Eye Hospital, Ahmedabad, India; ^2^Sudhalkar Eye Hospital and Retina Centre, Baroda, India

## Abstract

**Purpose:**

To compare the outcomes of trabeculectomy using two different routes of bevacizumab administration as an adjunct in patients with primary open angle glaucoma.

**Methods:**

Prospective, randomized, masked trial that included 180 eyes of 180 patients of documented primary open angle glaucoma were eligible for surgery. Patients were randomized to receive either a single intraoperative dose of subconjunctival bevacizumab (1.25 mg, Group I) or topical bevacizumab (5 mg/ml) for 30 days (Group II). One eye was randomly selected, if both were eligible for surgery. All patients underwent a complete ocular and systemic examination. Bleb morphology was examined and scored as per Moorfields system (MBGS) at 1, 3, 6, 12, 18, and 24 months postoperatively. Visual field, fundus photography, and disc analysis were performed. Outcome measures (at one year) included (1) comparison of bleb morphology in both groups, (2) proportion of patients achieving surgical success, and (3) side effects of treatment.

**Results:**

The groups did not differ with respect to age, sex, and crystalline lens status. Group II patients had significantly lower vascularity scores for central (*P*=0.042) and peripheral bleb areas (*P*=0.042) and peripheral bleb areas (*P*=0.042) and peripheral bleb areas (*n* = 88) patients achieved average vascular scores of less than 2.5 (*P*=0.042) and peripheral bleb areas (*n* = 88) patients achieved average vascular scores of less than 2.5 (*vs*. 94%; *P*=0.042) and peripheral bleb areas (

**Conclusion:**

Topical bevacizumab gives a better vascularity profile at one year, but the studied routes appear equally safe and do not seem to affect the outcome in any other way.

## 1. Introduction

Trabeculectomy is a standardized surgical procedure designed to lower intraocular pressure (IOP) and prevent or at least lessen glaucoma progression through the creation of an alternate drainage pathway for aqueous time [1, 2]. However, trabeculectomy may fail to lower IOP sufficiently in a sizable proportion of eyes. While some authors have reported varying degrees of success in trabeculectomy without the use of wound, mitomycin C (MMC) or 5-fluorouracil (5-FU) [3] has become somewhat of a routine in an attempt to prevent long-term surgical failures, with fibroblastic proliferation being the primary site of action. Although there are reports of good surgical success with these agents, their action is nonspecific, and various potentially sight-threatening complications [4, 5] are known to occur subsequent to their use. Thus, the current focus of interest is in the development of a safer and predictable adjuvant with a targeted focus of action for trabeculectomy, thereby increasing surgical success and minimizing complications [6].

The role of the vascular endothelial growth factor (VEGF) in various ocular pathophysiologic states is presently under intense research [7–13]. Its role in healing is well documented as is the fact that VEGF levels remain elevated postsurgery for nearly a month. VEGF levels are known to increase in the aqueous humour of patients with open-angle glaucoma (POAG) [7]. Bevacizumab is a 150kD full-length recombinant humanized monoclonal antibody that binds to all isoforms of vascular endothelial growth factor (VEGF) and is said to possess antiproliferative and antifibroblastic properties. Bevacizumab has been explored previously as an adjunct to trabeculectomy using different anti proliferative agents regarding the preferred route or dose of administration [7–10]. We aimed to determine the outcomes of the use of bevacizumab as an adjunct to trabeculectomy in primary open-angle glaucoma using two different routes of administration, namely, subconjunctival and topical.

## 2. Methods

This prospective, randomized, double-masked study was carried out at the Eye Hospital and Retinal Laser Centre, Baroda. Approval was obtained from the Institutional Review Board. The study conformed to the tenets of Helsinki and is registered at http://www.clinicaltrials.gov (unique protocol ID: NCT01425112). Informed consent was obtained from the patients at the entry level, with detailed counseling regarding the procedure, the potential sequelae, the off-label use of bevacizumab, the experimental nature of the study and the risks and benefits of conventional antimetabolites. Patients were recruited upon the requirement of trabeculectomy for open-angle glaucoma, with informed consent. For inclusion, patients were required to have a diagnosis of POAG, visual field, or optic disc changes characteristic of glaucoma and documented progression despite being on maximally tolerated medical therapy or progression due to noncompliance. Patients excluded were those diagnosed to have any other form of glaucoma, those with a systemic contraindication to bevacizumab, and those with an eye disease deemed to confound the diagnosis, analysis, and treatment of open-angle glaucoma, such as severe pathological myopia, complicated pseudophakia, aphakia, uveitis, proliferative diabetic retinopathy, or ocular neovascularization. A treatment history within the past three months, such as that of past conjunctival incisional surgery (including past filtering surgery), cataract surgery, detachment surgery, vitrectomy, or laser treatment of the eye also resulted in exclusion from the study; however, uncomplicated cataract surgery performed more than three months ago was not a contraindication to inclusion. A pilot study was conducted to determine the requisite sample size. Patients with primary open-angle glaucoma eligible for trabeculectomy (ten in each group) were randomized to receive either a single dose of subconjunctival bevacizumab (1.25 mg) intraoperatively or postoperative topical bevacizumab (5 mg/ml) over one month, and the patients followed up for six months. A difference of 30% was noted in the proportion of patients achieving an average vascularity score of less than 2.5 (an average of the scores for the vascularity of the central bleb area, peripheral bleb area, and peripheral nonbleb area) at the end of six months. More patients in the topical group achieved the said score (7 patients in the topical group versus 4 in the subconjunctival group). A significant reduction in the IOP was noted in both groups, and the drop persisted till the end of the follow-up period. No adverse events were noted in either group. However, this observed difference between the two groups could have been due to chance. For the current study, the null hypothesis stated there was no difference between the two groups. The current study, considering logistics, was designed to detect approximately an 8% difference in the proportion of patients achieving an average vascularity score of less than 2.5 at six months between the two groups. A total of 170 patients were estimated to be required for a 5% type I error and a 10% type II error (90% power). Primary analyses were done on an “intent to treat” basis.

### 2.1. Randomization and Masking

**Figure 1:**
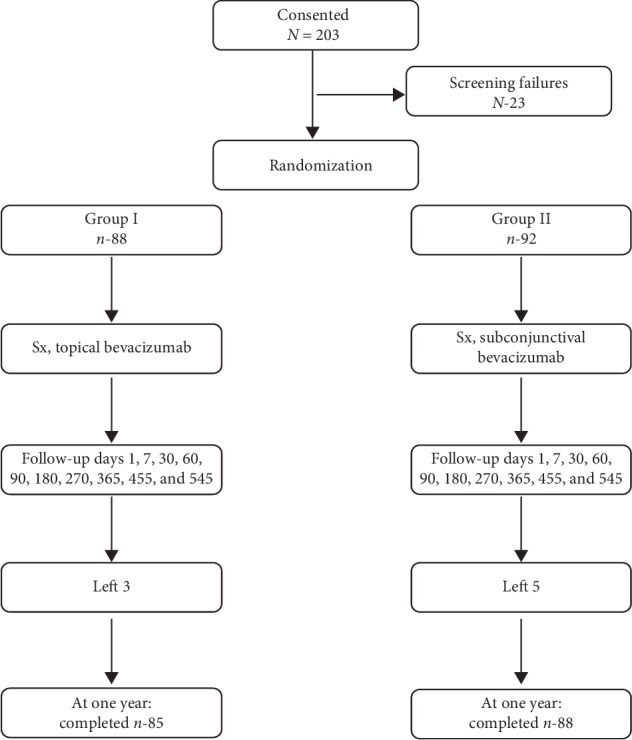
Flow chart for the study.

A single surgeon performed all procedures. Two different statisticians were employed for this study: one, to generate randomization protocols preoperatively, and the other, for postoperative analysis. The statistical analyst and the glaucoma expert who analyzed all patients postoperatively were masked to the study groups and objectives. However, a different statistician generated the preoperative randomization protocol, thereby ensuring he was not involved in the postoperative analysis, and hence there was no bias. Patients were randomly assigned (Figure 1) to receive either a single dose of subconjunctival bevacizumab (1.25 mg), intraoperatively, immediately upon completion of surgery (Group I) or topical bevacizumab (5 mg/ml) three times a day for 30 days (for an approximate total bevacizumab dose of 22.4 mg), starting postoperative day 2 (Group II). Randomization was achieved with the help of a computer-generated random number table, by a statistical analyst on the day of surgery. One eye of each patient was randomly selected for the study, again as determined by a statistician. The surgeon was informed of the group, a particular patient belonged to, intraoperatively, only towards at the end of surgery, after the completion of conjunctival suturing, to reduce the chance of bias in the surgical procedure. A trained assistant (who was informed of the group, a particular patient belonged to, only towards the end of surgery as well) would prepare bevacizumab (subconjunctival or topical) under validated aseptic conditions intraoperatively and hand over the subconjunctival injection to the surgeon (the patient should belong to the subconjunctival group) only at the end of conjunctival suturing. Thus, while nonviolation of the protocol was ensured, surgical time was not prolonged. Dilution of bevacizumab was achieved with sterile normal saline, and for topical use, bevacizumab was transferred to a sterile eye drops amber glass bottle. This was sealed with a sterile dropper standardized to deliver uniformly as per the metric drop system (1 ml = 20 drops). This was stored at 2°–8°C. Storage and dilution were as per company recommendations. The patients bore the cost of bevacizumab. The long-term stability and viability of bevacizumab, when stored in appropriate conditions is undisputed.. Any freshly opened bevacizumab bottle in our institute is used for no longer than one month; they are stored for particular duration in appropriate conditions with adequate asepsis.

### 2.2. Subclassification

Patients were classified into three subgroups for comparison between Groups I and II: (1) those who were phakic before trabeculectomy and remained so till the end of the follow-up period, (2) those who had both a visually significant cataract and glaucoma and had undergone uncomplicated cataract surgery (phacoemulsification) with in-the-bag IOL placement subsequent to the trabeculectomy, and (3) those who had already undergone uncomplicated temporal phacoemulsification with in-the-bag IOL placement at least three months prior to trabeculectomy.

### 2.3. Examination

All patients underwent a complete preoperative ocular examination including best corrected visual acuity, assessment of pupillary reaction, corneal pachymetry, slit-lamp examination (pre- and postdilatation), gonioscopy, applanation tonometry by Goldmann tonometer, optic disc examination and documentation (with *a* + 90D lens), and fundus examination which were done at each visit, and disc and RNFL photography using the Zeiss fundus camera and disc analysis using the VISUPAC software was performed every 6 months. Visual fields were recorded at baseline, prior to surgery and four months thereafter. Follow-ups were scheduled on postoperative days 1, 7, 30, 90, 180, 270, 365, and 730, with more frequent follow-ups implemented, and complications should necessitate the same. Following cataract surgery, patients were called on postoperative days 1 and 15, and the remaining follow-ups were made to coincide with posttrabeculectomy follow-ups, unless complications necessitated frequent monitoring.

### 2.4. Surgical Procedure

A single surgeon performed all surgeries in both groups using a standardized surgical technique. Intravenous mannitol (20%) was administered if required. Pupillary constriction was achieved with 2% pilocarpine eye drops. Trabeculectomy was performed superionasally. Following peribulbar anaesthesia and massage to reduce intraocular pressure, a fornix-based conjunctival flap was created with conjunctival scissors. A triangular flap, approximately half the scleral thickness was dissected in the sclera and raised. Dissection was carried out with the help of a crescent knife (Alcon Inc. Fort Worth, TX), a little into the clear corneal region immediately adjacent to the limbus to ensure that the entire trabecular area was exposed. A stab incision was made into the anterior chamber using a 15 degree sideport (Alcon Inc.), and a Kelly's punch was used to remove a part of the meshwork and create a window, approximately 1.5 × 1.5 mm. We aimed for consistency in the size of the opening in all patients. The excised tissue was examined on-table itself to ensure a part of the trabecular meshwork has been removed and identified by the typical structure under the microscope. A surgical peripheral iridotomy was created, using Vannas scissors, and the scleral flap was sutured with 10-0 nylon. Conjunctival closure was achieved with continuous sutures with 10–0 nylon. Other antimetabolites were not used. In Group A, a single subconjunctival injection of reconstituted bevacizumab was administered immediately adjacent to the bleb using a tuberculin syringe and 30-gauge needle. A single drop of atropine (1%) was instilled into the operated eye immediately at the end of surgery. Patients in both groups were asked to instill antibiotic and steroid drops (ofloxacin 0.3% and dexamethasone 1%) twice on the same day after having demonstrated to them the technique of instillation and punctal occlusion. From the first postoperative day, antibiotic steroid eye drops q.d.s. were prescribed and tapered over four weeks. In Group B, topical reconstituted bevacizumab drops (0.4 mg/drop) t.d.s were started from the second postoperative day for a period of one month. Bleb massage was permitted, if required, on posttrabeculectomy days 1 and 7. Phacoemulsification, when deemed necessary, was performed temporally using a standardized technique under peribulbar anaesthesia, at least three months after trabeculectomy and at least two months after the last dose of topical bevacizumab had been administered, provided the patient should belong to the topical group. Care was taken not to injure the bleb or trabeculectomy area.

### 2.5. Surgical Success and Failure

IOP was measured by Goldmann applanation tonometry with the patient seated at the slit lamp. An average of three readings was noted in each patient. All patients underwent diurnal measurements prior to surgery, postsurgery, and semiannually. For patient convenience, diurnal measurements were taken at any point in time within a range of 10 days before and after the scheduled 6-month follow-up and considered as appropriate for that particular follow-up, as not all patients find it possible to get diurnal IOP measurements done on the very date that they are scheduled for. Treatment success at one year was defined as an IOP of 8 to 18 mmHg (inclusive), at the 12-month follow-up visit. Absolute surgical success was defined as a postoperative IOP between 8–18 mmHg inclusive, along with at least a 20% reduction in the IOP from baseline, without the use of topical antiglaucoma medications, at the one year follow-up. Qualified success was defined as achievement of the same with the use of one topical antiglaucoma medication. Failure was defined as the inability to meet the aforementioned criteria and/or development of sight-threatening complications such as hypotony maculopathy or endophthalmitis. Postoperative complications such as shallow anterior chambers or wound leaks and the requisite subsequent interventions were not deemed failures, while additional glaucoma control procedures were.

### 2.6. Bleb Morphology

Bleb morphology was determined using the Moorfields Bleb Grading classification and documented in a manner similar to that used by Grewal and associates [10].

### 2.7. Visual Field and Progression Analysis

Visual field examination was done preoperatively and repeated every four months postoperatively using the Octopus 301 perimeter glaucoma or macular strategy, wherever appropriate. We ensured consistency in the use of appropriate strategies. If a particular patient was to undergo phacoemulsification in the fourth month after trabeculectomy, the visual field was done one month after cataract surgery and four months thereafter. EyeSuite trend analysis software was used to detect progression, and the number of patients who demonstrated significant progression in each group postoperatively was noted.

#### 2.7.1. Safety Assessment

Safety assessments included detailed ophthalmic examinations such as slit-lamp biomicroscopy, visual acuity, visual field, funduscopy, cataract development as per Lens Opacities Classification System III (where applicable), uveitis (flare and cells), hypotony, bleb leaks, allergic reaction, unexplained poor vision, corneal changes, and retinal changes at each visit. Other safety assessments included physical examination, adverse events, laboratory tests (for hematology, urinary examination and biochemistry) on days 15, 22, and 30 after initiation of bevacizumab therapy. Any clinically significant abnormalities observed in the nonstudy eye were recorded. Patients with systemic disease were monitored in strict consort with a physician.

#### 2.7.2. Outcome Measures

The primary outcome measure was comparison of bleb morphology in both groups along with the proportion of patients achieving an average vascularity score of less than 2.5. Secondary outcome measures included the proportion of patients achieving surgical success (absolute and qualified) as well as the adverse events in both groups. Statistical analysis included Kolmogorov–Smirnov test for confirming the normality of the distribution, the paired and unpaired *t*-test, Mann–Whitney *U* test, Fisher's exact test, Wilcoxon test, and the *Z* test, wherever appropriate. Statistical analysis was performed using the SPSS 16 software (SSPSS Inc. Chicago, IL). Statistical significance was set at a value of *P* < 0.05.

## 3. Results

### 3.1. Demographics

**Figure 2:**
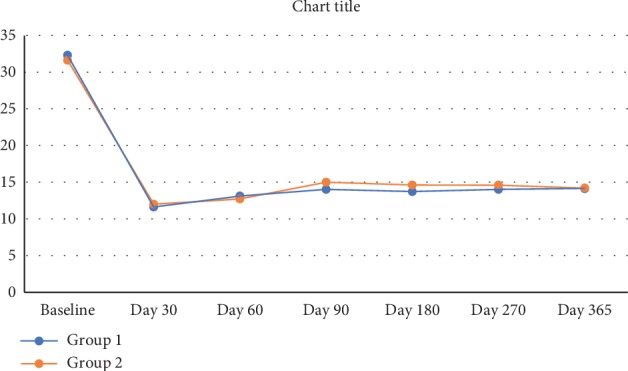
The mean IOP for all patients in both groups at each visit, along with the “*P*” values, demonstrating that there was no significant difference between the groups at any stage. 172 patients were available for all follow-ups up to one year. 12 patients in Group I and 14 in Group II completed two years of follow-up.

A total of 180 patients eventually qualified for the study, out of 203 patients who consented for the study. Eighty-five patients in Group I and eighty-eight patients in Group II were available for the one year follow-up, and as attrition and the number of available patients did not differ significantly between the groups, for the sake of uniformity, all descriptive statistics and figures refer to the findings at one year. There were 39 males and 46 females in Group I and 47 males and 41 females in Group II. The descriptive statistics for the subgroups in Groups I and II are outlined in Tables 1–3. The groups did not differ with respect to age, gender, and crystalline lens status. All patients had received at least two of the following topical antiglaucoma medications prior to surgery: beta-blockers, prostaglandin analogs, sympathomimetics and topical carbonic anhydrase inhibitors (after consideration of contraindications and tolerance). The Kolmogorov–Smirnov test confirmed the normality of the IOP distribution in both groups. There were seven diabetics and three hypertensives in Group A and eleven diabetics and five hypertensives in Group B, all well controlled, without any systemic disease. Two diabetics in Group A and three in Group B had minimal diabetic retinopathy as per the ETDRS classification, which did not warrant treatment. There were eleven patients in Group I and four in Group II who had an axial length of 26.0 mm or more (Fisher's exact test, *P* = 0.0511; not statistically significant). The mean follow-up was 15.25 ± 3.2 months in Group I and 16.00 ± 2.40 months in Group II. No adverse events were observed in the nonstudy eye in any patient in either group (Figure 2).

### 3.2. Intraocular Pressure and Visual Acuity (IOP)

**Figure 3:**
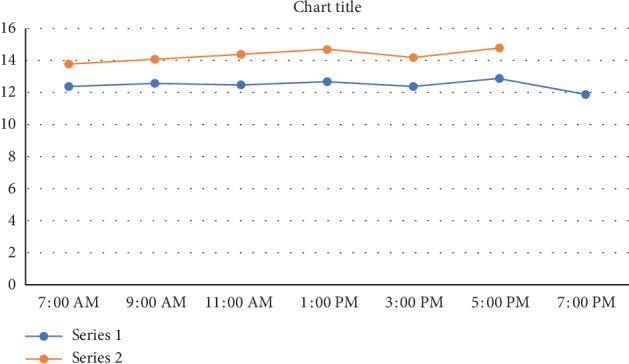
The mean diurnal IOP for all patients in both groups at the one year visit, with *P* values demonstrating no difference between the two groups. *X*-axis represents the time intervals at which IOP was measured. *Y*-axis represents the intraocular pressure (IOP) at a given point in time.

**Figure 4:**
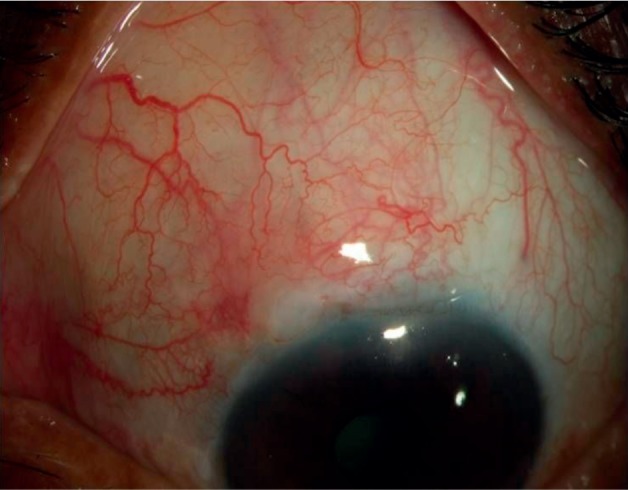
A diffuse slit-lamp image of a patient from Group I at one year of follow-up.

**Figure 5:**
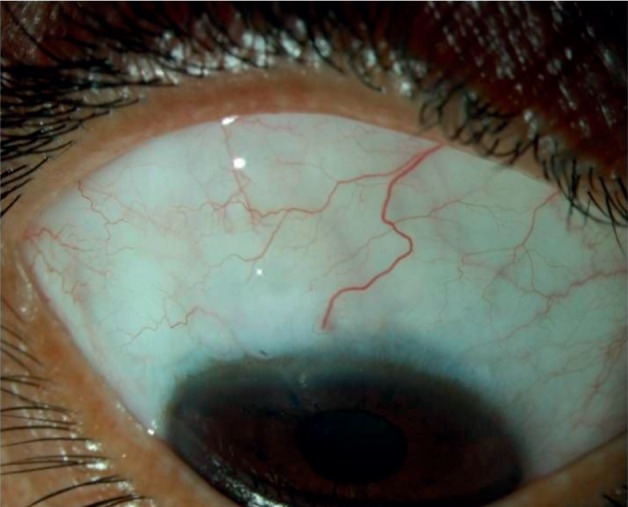
A diffuse slit-lamp image of a patient from Group II at one year of follow-up.

A significant reduction was observed in the IOP posttrabeculectomy (Tables 1–3, Figure 3) in all patients in both groups (paired *t*-test, *P* < 0.001, both groups), a change that had persisted at the one year follow-up. The number of patients who achieved absolute and surgical success is shown in Table 4. Surgical success (absolute and qualified combined) did not vary significantly amongst the groups (96% vs. 94%). The amplitude of diurnal IOP fluctuations (Tables 1–3) did not change significantly before and after trabeculectomy (Wilcoxon rank test: Group I, *P*=0.54; Group II, *P*=0.43). The number of postoperative medications (Tables 1–3) was significantly reduced in both groups posttrabeculectomy as compared with the preoperative status (Wilcoxon rank test: *P* < 0.001, both groups). Visual acuity (Tables 1–3) did not change significantly before and after trabeculectomy in subgroup 1 (Wilcoxon rank test: Group I, *P*=0.54; Group II, *P*=0.60) and subgroup 3 (Group I, *P*=0.43; Group II, *P*=0.56). The same was true for subgroup II (Wilcoxon rank test: Group I, *P*=0.24; Group II, *P*=0.27) after trabeculectomy but improved significantly after cataract extraction (Wilcoxon rank test: Group I, *P*=0.045; Group II, *P*=0.038). Beta-blockers were used if required for IOP control after surgery. It was contraindicated in two patients in Group I, who received sympathomimetics instead. There was no clinically significant difference between the two groups in terms of the mean IOP at any of the visits (Figure 3), as well as the diurnal IOP at the one year visit. Survival analysis, using the criteria of absolute and qualified success, failed to show a difference between the two groups and demonstrated an almost identical response to treatment. Three patients in Group I and five in Group II failed as per our earlier definition. Of these, two patients in Group I and four in Group II required additional surgery for IOP control and received topical and subconjunctival bevacizumab, respectively, as adjuvants. One patient each in both groups required more than one antiglaucoma medication for IOP control after trabeculectomy (Figures 4 and 5).

### 3.3. Bleb Morphology

Patients in the topical group showed a better vascularity score as compared with the subconjunctival group in all three subcategories of vascularity at the one year follow-up. This difference was not quite significant at the two year follow-up. In all other respects, the bleb characteristics were not significantly different between the two groups (Table 5). 68% of the topical group achieved an average vascularity score of less than 2.5 as compared with 45% in the subconjunctival group at one year.

### 3.4. Visual Field Analysis

The average preoperative mean deviation was not significantly different between the groups: −9.32 ± 4.21 dB in Group I and −11.41 ± 4.05 dB in Group II (Wilcoxon rank test: *P*=0.42). The average postoperative mean deviation in Group I was −9.57 ± 4.17 and −11.59 ± 5.13 dB in Group II. The difference at one year was statistically insignificant (Wilcoxon rank test: *P*=0.74 in Group I; *P*=0.71 in Group II) at one year. There was no statistically significant difference in the mean deviation between both groups at one year (Wilcoxon rank test:*P*=0.32). The average corrected loss variance (cLV) in Group I was −6.43 ± 3.54 dB preoperatively and −6.31 ± 2.76 dB postoperatively at one year (Wilcoxon rank test: *P*=0.87). The average cLV in Group II was −5.0 ± 4.32 preoperatively and −5.91 ± 4.40 postoperatively at one year (Wilcoxon rank test: *P*=0.70). The difference between the two groups was statistically insignificant both preoperatively (Wilcoxon rank test: *P*=0.45) and postoperatively (Wilcoxon rank test: *P*=0.38). None of the patients showed significant progression at one year, but the results would obviously be confounded by there being insufficient data points for appropriate analysis at one year.

### 3.5. Cataract Extraction

Table 2 lists the descriptive statistics of patients who underwent uncomplicated temporal phacoemulsification and in the bag IOL implantation at least three months after the trabeculectomy. This includes two patients in Group I and one in Group II who developed cataract as a complication of trabeculectomy. All surgeries were uneventful. The mean time to cataract extraction was 120.45 ± 32.64 days after trabeculectomy in Group I, and 132.54 ± 24.44 days in Group II (Wilcoxon test: *P*=0.26). The time to cataract development in the two patients in Group I (consequent to the trabeculectomy) was six and nine months after trabeculectomy, respectively. The solitary patient in Group II who developed cataract did so three months after trabeculectomy. All had clear lenses prior to trabeculectomy, but showed cataract development after trabeculectomy at the stated time periods. All three had uneventful cataract surgery.

### 3.6. Adverse Events and Failures

There was no statistically significant difference between the two groups in terms of the number of patients who failed (Table 4), or the number of patients with complications (Table 6). 4 eyes in Group I and 3 in Group II had subconjunctival blood more than the scleral flap, but it resolved in all without sequel. Group I showed a slightly higher incidence of a patch of subconjunctival hemorrhage (11 eyes) as compared with Group II (5 eyes); the difference was not statistically significant (*P*=0.103). The subconjunctival hemorrhage in these cases was smaller than the scleral flap in all cases and resolved in all patients. Wound leaks responded to resuturing, and only one patient in Group I had a flat anterior chamber required air injection on postoperative day 1. The other patients in both groups with a shallow anterior chamber were monitored, but chamber depth improved by day 3 by patching the eye for two days. No patient in either group developed major complications such as endophthalmitis, scleral thinning or perforation, vitreous or systemic hemorrhage, ocular surface inflammation, choroidal detachments, or hypotony maculopathy. One hypertensive patient in Group II required additional antihypertensive medication from posttopical bevacizumab therapy on day 15, for the remainder of the duration of topical bevacizumab therapy, which could be discontinued one week after the first postoperative month, i.e., one week after cessation of topical bevacizumab therapy. He did not require any additional medication ever after, and we therefore presumed that the increase in blood pressure was bevacizumab-induced. Laboratory abnormalities and various signs and symptoms noted in both groups are recorded in Table 7; topical therapy was not required to be discontinued in any of the patients, and none of the patients from the subconjunctival group required additional therapy for treatment of signs and symptoms. None of the patients in either group had any complaints or showed laboratory abnormalities before day 15 postbevacizumab therapy initiation.

## 4. Discussion

This study demonstrates very good results in a relatively healthy population with primary open angle glaucoma using two convenient modes of bevacizumab administration with minimal complications. Additionally, we had excellent compliance with respect to follow-ups at one year, thus making our analysis uniform and informative. All patients were well educated, with all having at least graduated from college, and this can probably influence compliance as well. Our results in terms of surgical success are comparable with the Hitchings follow-up, and there was no clinically significant difference between the two groups in terms of IOP control and failure rates. Diurnal IOP amplitudes did not show significant differences before and after trabeculectomy in either group probably because all patients were already under medical therapy, although absolute IOP values showed significant reduction. Though there appears to be a trend of increase in IOP from baseline to the one year follow-up, there was no significant difference between postoperative IOP on day 1 and day 365. Visual acuity too did not deteriorate, and actually improved in the group which received cataract surgery. Visual fields did not progress, but analysis at one year is too short a period to comment on the same. The importance of determining the appropriate route of administration for bevacizumab has been emphasized in recent secondary open-angle glaucoma (pseudoexfoliation) patients, whereas our study was based exclusively on primary open-angle glaucoma patients. Patients undergoing additional surgery too did not show any adverse events despite additional bevacizumab use. Starting topical bevacizumab from day 2 coincides with peak VEGF bevacizumab and has been suggested to delay wound healing in studies using subconjunctival bevacizumab (e.g., in the study by Grewal and associates, high myopes were poorly represented in either group, especially Group II, but none of them showed adverse events). The role of bevacizumab in trabeculectomy has been explored in several earlier studies [13]. No patients demonstrated significant local or systemic adverse events. Bevacizumab administration thus appeared safe both locally and systemically. The abnormalities in findings noted in both groups were not significantly different. Topical bevacizumab, probably on account of prolonged administration or a higher cumulative dose, resulted in better individual and average vascularity. The topical group, despite having received a higher cumulative dose on the ocular surface, and probably systemically as well, did not show a higher rate of complications.

This study is not without limitations. The most obvious of those would be that it does not afford a comparison with established adjuvant therapy, such as with MMC or 5-FU. The exact dose administered in the topical group would vary from patient to patient, despite strict instructions on compliance, storage, regular medication usage checks, involvement of relatives, and punctal occlusion. We did not explore the option of multiple doses, and one cannot really expect to detect significant visual field progression in one year, especially with some patients already advanced in glaucomatous damage. Also, though no systemic adverse events were noted in any patient in either group, we did not assess systemic levels of bevacizumab. This study, however, helps compare the role of bevacizumab as an adjunct to trabeculectomy and two different routes of administration. This study, to the best of our knowledge, is the first that provides comparative details of the subconjunctival and topical route. Subconjunctival administration can nullify the issue of compliance, variability in dosage as well as convenience, factors which are crucial to the success of topical therapy. Future studies with larger sample sizes could possibly look at other routes of administration, at its role in phacotrabeculectomy and at direct comparisons with or without antimetabolites for trabeculectomy in open angle glaucoma. The use of other anti-VEGF agents seems to result in better, diffuse and avascular blebs as current study, as well as per past seem to significantly affect the outcome (except in terms of vascularity), at least till the end of one year. To the best of our knowledge, this is the largest series to date on the use of bevacizumab in open-angle glaucoma.

## Figures and Tables

**Table 1:** Phakic patients: descriptive statistics.

Group I age	Group II age	*P* value	Number of patients	Group I	Group II	

58.24 years ± 9.24 years	56.32 ± 11.1 years	0.48	*N* = patients	56	49	

Parameter	Preoperative value		*P* value	Postoperative values		*P* value
	Group I	Group II		Group I	Group II	

IOP (mm Hg)	30.5 ± 4.0	29.3 ± 3.11	0.87	15.34 ± 2.34	14.10 ± 3.20	0.58
CDVA (logMAR)	0.48 ± 0.31	0.42 ± 0.24	0.72	0.44 ± 0.25	0.40 ± 0.26	0.42
Diurnal IOP	4.47 ± 2.32	3.50 ± 1.40	0.77	2.13 ± 0.89	1.72 ± 0.5	0.56
Number of medications	3.22 ± 1.1	3.10 ± 1.48	0.85	0.11 ± 0.04	0.12 ± 0.03	0.54
Duration of medicine use	6.35 ± 4.25 years	4.56 ± 5.23 years	0.32			

^*∗*^Group I: 24 males and 32 females; Group II: 29 males and 20 females.

**Table 2:** Posttrabeculectomy cataract surgery patients: descriptive statistics.

Group I	Group II	*P* value	Number of patients	Group I	Group II	

63.21 ± 9.24 years	65.42 ± 11.1 years	0.59	*N* = patients	21	27	

Parameter	Preoperative value		*P* value	Postoperative value		*P* value
Groups	Group I	Group II		Group I	Group II	

IOP	28.42 ± 4.20	27.6 ± 5.00	0.62	14.34 ± 2.34	15.10 ± 3.30	0.83
CDVA	0.54 ± 0.36	0.46 ± 0.14	0.24	0.30 ± 0.25	0.24 ± 0.20	0.28
Diurnal IOP	3.87 ± 3.31	3.77 ± 1.42	0.80	2.77 ± 0.69	2.82 ± 1.0	0.82
Number of medications	2.96 ± 1.21	2.82 ± 1.6	0.78	0.09 ± 0.04	0.13 ± 0.09	0.72
Duration of medicine use	4.14 ± 4.0 years	4.14 ± 4.0 years	0.46			

Group I: 11 males and 10 females; Group II: 11 males and 16 females.

**Table 3:** Pseudophakic patients: descriptive statistics.

Group I age	Group II age	*P* value	Number of patients	Group I	Group II	

69.21 ± 5.32 years	65.12 ± 4.3 years	0.48	*N* = patients	8	12	

Parameter	Preoperative value		*P* value	Postoperative value		*P* value
Groups	Group I	Group II		Group I	Group II	

IOP (mm Hg)	25.42 ± 5.20	28.6 ± 6.90	0.62	13.34 ± 2.34	14.10 ± 3.20	0.83
CDVA (logMAR)	0.34 ± 0.31	0.34 ± 0.24	0.94	0.36 ± 0.25	0.30 ± 0.26	0.71
Diurnal IOP	3.11 ± 2.11	1.53 ± 1.42	0.91	1.33 ± 0.69	1.82 ± 1.0	0.78
Number of medications	2.96 ± 1.21	2.82 ± 1.6	0.86	0.05 ± 0.03	0.08 ± 0.09	0.90
Duration of medicine use	4.14 ± 4.0 years	5.75 ± 4.15 years	0.43			

Group I: 4 males and 4 females; Group II: 7 males and 5 females.

**Table 4:** Success rate at one-year follow-up.

Groups	Absolute success	Qualified success	Failure
Group I	72 (78.26%)	10 (18.22%)	3 (3.52%)
Group II	71 (83.43%)	12 (10.9%)	5 (5.68%)
*Z*-test	*P*=0.47	*P*=0.267	*P*=0.51

**Table 5:** Complications.

Groups	Shallow A/C	Leaking bleb	Ocular surface problems	Cataract formation	Others
Group I	2 eyes	1 eye	3-dry eyes	2	0
Group II	3 eyes	1	5-dry eyes	1	0

**Table 6:** MBGS scores and “*P*” values for all subcategories at one-year follow-up.

MBGS subcategories	Group I (scores) 3.3 ± 1.1 2.60 ± 1.32 3.2 ± 1.2 3.7 ± 0.47 3.74 ± 0.88 1.6 ± 0.41 *n* = 4 eyes	Group II (scores) 3.21 ± 0.89 3.1 ± 0.79 3.7 ± 1.0 1.67 ± 0.42 1.89 ± 0.79 3.4 ± 0.77 *n* = 3 eyes	“*P*” value *P*=0.34 *P*=0.17 *P*=0.32 *P*=0.042 *P*=0.023 *P*=0.03 *P*=0.30
(1a) Central maximal area			
(1b) Entire maximal area			
(2) Bleb height			
(3a) Vascularity of central demarcated area of bleb			
(3b) Vascularity of peripheral area			
(3c) Peripheral nonbleb area			
Subconjunctival blood present (greater than scleral flap)			

**Table 7:** Laboratory parameters.

Abnormal laboratory investigations/physical examination	Group I	Group II
Urinary protein positive^*∗*^	5	8
Prolonged prothrombin time^*∼*^	1	1
Decreased white blood cell _count_^*∗∗*, *∼*^	1	3
Nausea	4	2
Diarrhea	0	1
Fatigue	2	1
Mild abdominal pain	1	1
Myalgia	3	2

^*∗*^Noticed on days 22 and 30 postinitiation; ^*∗∗*^the decrease was less than 20% in all four cases, and in none of the patients did the count go below 4000 cells/mm [3]. ^*∼*^Noticed on day 30 postinitiation of bevacizumab therapy.

## Data Availability

The data used to support the findings of this study are available from the corresponding author upon request.
